# Validation and refinement of a predictive nomogram using artificial intelligence: assessing in-hospital mortality in patients with large hemispheric cerebral infarction

**DOI:** 10.3389/fneur.2024.1398142

**Published:** 2024-06-25

**Authors:** Jian Ding, Xiaoming Ma, Wendie Huang, Chunxian Yue, Geman Xu, Yumei Wang, Shiying Sheng, Meng Liu, Yi Ren

**Affiliations:** ^1^Department of Neurology, The Third Affiliated Hospital of Soochow University, Changzhou, China; ^2^Department of Neurology, Suzhou Hospital, Affiliated Hospital of Medical School, Nanjing University, Suzhou, China; ^3^Department of Neurology, Affiliated Fuyang People’s Hospital of Anhui Medical University, Fuyang, China

**Keywords:** nomogram, cerebral infarction, prognosis, mortality, stroke, ROC curve

## Abstract

**Background:**

Large Hemispheric Infarction (LHI) poses significant mortality and morbidity risks, necessitating predictive models for in-hospital mortality. Previous studies have explored LHI progression to malignant cerebral edema (MCE) but have not comprehensively addressed in-hospital mortality risk, especially in non-decompressive hemicraniectomy (DHC) patients.

**Methods:**

Demographic, clinical, risk factor, and laboratory data were gathered. The population was randomly divided into Development and Validation Groups at a 3:1 ratio, with no statistically significant differences observed. Variable selection utilized the Bonferroni-corrected Boruta technique (*p* < 0.01). Logistic Regression retained essential variables, leading to the development of a nomogram. ROC and DCA curves were generated, and calibration was conducted based on the Validation Group.

**Results:**

This study included 314 patients with acute anterior-circulating LHI, with 29.6% in the Death group (*n* = 93). Significant variables, including Glasgow Coma Score, Collateral Score, NLR, Ventilation, Non-MCA territorial involvement, and Midline Shift, were identified through the Boruta algorithm. The final Logistic Regression model led to a nomogram creation, exhibiting excellent discriminative capacity. Calibration curves in the Validation Group showed a high degree of conformity with actual observations. DCA curve analysis indicated substantial clinical net benefit within the 5 to 85% threshold range.

**Conclusion:**

We have utilized NIHSS score, Collateral Score, NLR, mechanical ventilation, non-MCA territorial involvement, and midline shift to develop a highly accurate, user-friendly nomogram for predicting in-hospital mortality in LHI patients. This nomogram serves as valuable reference material for future studies on LHI patient prognosis and mortality prevention, while addressing previous research limitations.

## 1 Introduction

Large Hemispheric Cerebral Infarction (LHI) is a severe subtype of ischemic stroke, often accompanied by complications such as cerebral edema, pneumonia, and infarct transformation hemorrhage (1). LHI typically refers to a complete or partial anterior circulation infarction resulting from proximal occlusion of the internal carotid artery or the middle cerebral artery (MCA). It constitutes approximately 10% of all ischemic strokes, making it one of the most validated subtypes of ischemic stroke (2). Simultaneously, it is characterized by a heightened risk of in-hospital mortality, ranging from 30 to 80%. Given the complexity of LHI and its associated mortality risk, there is an imperative need to develop predictive models that facilitate early identification of patients at higher risk, enabling timely interventions and personalized care strategies. Previous study (3) had highlighted the potential benefits of decompressive hemicraniectomy (DHC), reporting a significant improvement in survival rates ranging from 67 to 84%. However, the limited acceptance of DHC can be attributed to its stringent eligibility criteria. Another clinical trial (4) has demonstrated the effectiveness of intravenous glyburide in reducing midline shift and mortality associated with edema in patients with large hemispheric infarction (LHI), though it did not mitigate the risk of malignant brain edema or improve 3-month functional outcomes. Despite the intricate nature of early mortality causes in LHI patients, current understanding of the treatment plan remains the needing of further research.

Given the intricate nature of LHI and its’ heightened mortality risk, this study aims to proactively employ the Boruta artificial intelligence algorithm in conjunction with Logistic Regression, based on a retrospective investigation. The objective is to construct a predictive model for in-hospital mortality risk among LHI patients, facilitating real-time assessment during their hospitalization and offering valuable guidance for clinical decision-making. Furthermore, the study seeks to externally validate and propose more pragmatic enhancements to the previous research (5). The ultimate goal is to propose more practical improvements to existing researches endeavors.

## 2 Methods

### 2.1 Participants

Patients diagnosed with LHI at the Department of Neurology in the Third Affiliated Hospital of Soochow University between December 2018 and April 2023 were included in this study. The study received ethical approval from the Ethics Review Board of the Third Affiliated Hospital of Soochow University (Approval Number: 2023-S-080). Due to the retrospective nature of the study, the requirement for obtaining informed consent from the participants was waived.

The inclusion criteria utilized in this study are consistent with those of a prior investigation (6), in order to maintain standard uniformity in addressing the MCE issue:

1. Diagnosis of acute ischemic stroke
2. Lesion site included the blood supply area of the MCA with or without additional affected regions, and the cerebral infarction area was at least two-thirds of the blood supply area of the MCA
3. Age ≥ 18 years
4. Time from onset to admission <72 h
5. Vascular recanalization therapy, such as thrombectomy or thrombolysis, was not administered.

Patients who previously experienced severe organ dysfunction or were diagnosed with major medical conditions, including cancer, were excluded from the study.

### 2.2 Data collection and group division

A comprehensive dataset was constructed, encompassing demographic characteristics, clinical variables, vascular risk factors, and laboratory findings. Although a few pieces of information were found to be missing, the proportion of missing data remained within an acceptable range (less than 30%). To address this limitation, Multiple Imputation by Chained Equations was applied separately for both patient groups, which had been previously categorized based on the occurrence of in-hospital death to prevent intergroup data feature leakage. These imputation techniques were used to accurately fill in the missing data, ensuring a more robust and complete analysis.

Subsequently, the entire population was randomly divided into the Development Group and Validation Group at a ratio of 3:1. The inter-group comparison of the categorical variables was conducted with the continuous correction chi-square test, while the continuous variables were compared via one-way ANOVA. No statistically significant differences were existed in all the variables and outcome between the Development Group and the Validation Group. Due to the large population distribution, it is mandatory to use normal distribution to present continuous data. Categorical data consists of composition ratios, also, the Details can be found in the Supplementary Table 1. This study was approved by the Ethics Review Board of the Third Affiliated Hospital of Soochow University (Approval Number: 2023-S-080). Due to the retrospective nature of the study, the requirement of obtaining informed consent from the participants was waived.

### 2.3 Imaging evaluation

Midline shift, as well as non-MCA territory involvement, which pertains to the additional inclusion of the anterior and/or posterior cerebral artery territory, were independently evaluated by two experienced physicians with over a decade of clinical practice. Precise measurements in millimeters were recorded for midline shift. In cases of discrepancies, a third senior physician provided resolution.

### 2.4 Variable selection

Using the Bonferroni-corrected Boruta technique (7), a wrapper-based feature selection method, variables were screened across the entire dataset to retain a subset of features correlated with the dependent variable. Throughout this process, the significance level for *p*-values was set at 0.01 to ensure the selected features possess a high degree of statistical significance.

### 2.5 Logistic regression and nomogram development

Initially, all 15 variables retained by the Boruta algorithm were included in the Logistic Regression. Subsequently, a backward stepwise regression method was employed to iteratively refine the model and reduce its Akaike Information Criterion (AIC) value. Finally, the variables NIHSS score, Collateral Status, NLR, Ventilation, Midline shift, Neutrophil-to-lymphocyte ratio, and APACHE II Score were retained. Variance inflation factor tests were conducted to assess the presence of multicollinearity among variables in the logistic regression, finally nomogram was constructed.

Meanwhile, ROC curve and DCA curve were drawn according to the Nomogram model. Calibration technique was performed based on the data from Validation Group.

### 2.6 Comparing two models

Utilizing the Net Reclassification Improvement (NRI) and Integrated Discrimination Improvement (IDI) metrics, along with DCA curves, aiming to compare the performance of the two models and assess their strengths and weaknesses. The NRI and IDI metrics provide quantitative measures for the improvement in risk prediction achieved by one model over another. By incorporating these indices and examining DCA curves, we can comprehensively evaluate and compare the efficacy of the two models in predicting outcomes. This integrated approach will offer insights into the superiority or inferiority of each model, aiding in a more robust assessment of their predictive capabilities.

## 3 Results

### 3.1 Demographic characteristics and clinical information

In this study, a total of 314 patients with acute anterior circulating Large Hemispheric Infarction (LHI) were recruited, with 29.6% of the patients assigned to the Death group (*n* = 93). The baseline characteristics and clinical information of the patients are presented in Table 1.

**Table 1:** Demographic characteristics and clinical information of the study patients (*n* = 314).

Characteristics	All (*n* = 314)	Survival group (*n* = 221)	Death group (*n* = 93)	*p*-value
Age	75.67 ± 12.28	75.10 ± 12.12	77.02 ± 12.62	0.207
Glascow coma score	9.90 ± 3.56	10.82 ± 3.37	7.71 ± 3.04	<0.001
ASPECTS	5.32 ± 3.08	6.05 ± 2.75	3.56 ± 3.12	<0.001
NIHSS score	16.81 ± 6.56	14.93 ± 5.93	21.28 ± 5.79	<0.001
collateral score	1.49 ± 1.08	1.71 ± 1.00	0.95 ± 1.06	<0.001
WBC	28.93 ± 74.04	26.48 ± 73.38	34.75 ± 75.65	0.366
Neutrophil count	8.91 ± 3.67	8.18 ± 3.23	10.66 ± 4.06	<0.001
Lymphocyte count	1.77 ± 2.57	1.76 ± 2.22	1.82 ± 3.28	0.854
NLR	9.20 ± 9.00	7.44 ± 5.85	13.39 ± 12.98	<0.001
Fibrinogen level	3.94 ± 2.33	3.83 ± 1.32	4.22 ± 3.76	0.166
SBP	149.77 ± 23.30	150.06 ± 22.14	149.08 ± 25.96	0.732
DBP	83.03 ± 14.93	83.04 ± 14.39	83.01 ± 16.23	0.987
APACHE II	13.20 ± 5.50	11.83 ± 4.97	16.46 ± 5.33	<0.001
Midline shift (mm)	3.41 ± 5.28	2.12 ± 3.54	6.49 ± 7.17	<0.001
Sex				
Female	156 (49.68)	109 (49.32)	47 (50.54)	0.942
Male	158 (50.32)	112 (50.68)	46 (49.46)	
TOAST classification				
Large-artery atherosclerosis	171 (54.46)	129 (58.37)	42 (45.16)	0.043
Cardioembolism	143 (45.54)	92 (41.63)	51 (54.84)	
Consciousness disorders				
No	110 (35.03)	96 (43.44)	14 (15.05)	<0.001
Yes	204 (64.97)	125 (56.56)	79 (84.95)	
History of hypertension				
No	72 (22.93)	44 (19.91)	28 (30.11)	0.069
Yes	242 (77.07)	177 (80.09)	65 (69.89)	
History of diabetes mellitus				
No	200 (63.69)	135 (61.09)	65 (69.89)	0.176
Yes	114 (36.31)	86 (38.91)	28 (30.11)	
Atrial fibrillation				
No	177 (56.37)	136 (61.54)	41 (44.09)	0.006
Yes	137 (43.63)	85 (38.46)	52 (55.91)	
Ventilation				
No	241 (76.75)	206 (93.21)	35 (37.63)	<0.001
Yes	73 (23.25)	15 (6.79)	58 (62.37)	
History of stroke				
No	226 (71.97)	157 (71.04)	69 (74.19)	0.667
Yes	88 (28.03)	64 (28.96)	24 (25.81)	
Smoking history				
No	256 (81.53)	177 (80.09)	79 (84.95)	0.394
Yes	58 (18.47)	44 (19.91)	14 (15.05)	
Drink history				
No	271 (86.31)	190 (85.97)	81 (87.10)	0.932
Yes	43 (13.69)	31 (14.03)	12 (12.90)	
Pneumonia				
No	97 (30.89)	69 (31.22)	28 (30.11)	0.951
Yes	217 (69.11)	152 (68.78)	65 (69.89)	
UTI				
No	283 (90.13)	196 (88.69)	87 (93.55)	0.267
Yes	31 (9.87)	25 (11.31)	6 (6.45)	
Gastrointestinal bleeding				
No	296 (94.27)	209 (94.57)	87 (93.55)	0.928
Yes	18 (5.73)	12 (5.43)	6 (6.45)	
Hemorrhagic transformation				
No	236 (75.16)	162 (73.30)	74 (79.57)	0.303
Yes	78 (24.84)	59 (26.70)	19 (20.43)	
Seizure				
No	303 (96.50)	213 (96.38)	90 (96.77)	1
Yes	11 (3.50)	8 (3.62)	3 (3.23)	
Admission anisocoria				
No	263 (83.76)	198 (89.59)	65 (69.89)	<0.001
Yes	51 (16.24)	23 (10.41)	28 (30.11)	
Admission Gaze deviation				
No	160 (50.96)	129 (58.37)	31 (33.33)	<0.001
Yes	154 (49.04)	92 (41.63)	62 (66.67)	
Lesion side				
Left	149 (47.45)	108 (48.87)	41 (44.09)	0.515
Right	165 (52.55)	113 (51.13)	52 (55.91)	
Infarction involving non-MCA Perfusion Territories				
No	196 (62.42)	152 (68.78)	44 (47.31)	0.001
Yes	118 (37.58)	69 (31.22)	49 (52.69)	
Lateral ventricular compression				
No	136 (43.31)	105 (47.51)	31 (33.33)	0.029
Yes	178 (56.69)	116 (52.49)	62 (66.67)	
Basalcistern effacement				
No	231 (73.57)	182 (82.35)	49 (52.69)	<0.001
Yes	83 (26.43)	39 (17.65)	44 (47.31)	

### 3.2 Boruta variable selection and logistic regression

After applying the Boruta algorithm for variable selection, a total of 15 variables were identified as significant. Which were Glasgow Coma Score, Collateral Score, White Blood Cell count, Neutrophil count, Lymphocyte count, NLR (Neutrophil-to-Lymphocyte Ratio), Ventilation, Non-MCA territorial involvement, Admission Diastolic Blood Pressure, Midline Shift, and Basal cistern, APACHE II score, NIHSS score, ASPECTS score, HbA1c.

All the 15 variables mentioned above were included in the logistic regression (Table 2), subsequently a stepwise backward elimination approach was employed to refine the model and reduce its AIC. The final model retained 6 variables: NIHSS score, Collateral Score, NLR, Ventilation, Non-MCA territorial involvement, and Midline Shift. Meanwhile, the model’s AIC decreased from 183.55 to 171.65.

**Table 2:** Univariate and multivariate logistic regression of the six predictors.

Variables	Univariate analysis			Multivariate analysis			
	OR	95% CI	*p*-value	OR	95% CI	*p*-value	VIF
NIHSS	1.23	1.16–1.31	<0.001	1.13	1.04–1.22	0.003	1.44
Collateral score	0.38	0.27–0.51	<0.001	0.63	0.41–0.95	0.031	1.35
NLR	1.08	1.04–1.13	<0.001	1.05	1.00–1.10	0.069	1.14
Ventilation							1.25
No	1.00 (ref)			1.00 (ref)			
Yes	18.14	8.77–40.08	<0.001	9.45	3.99–23.99	<0.001	
Infarction involving Non-MCA Perfusion Territories							1.06
No	1.00 (ref)			1.00 (ref)			
Yes	2.53	1.43–4.51	0.002	2.50	1.11–5.79	0.029	
Midline shift (mm)							1.25
No	1.00 (ref)			1.00 (ref)			
Yes	1.20	1.13–1.29	<0.001	1.12	1.04–1.22	0.004	

### 3.3 Nomogram assessment

**Figure 1:**
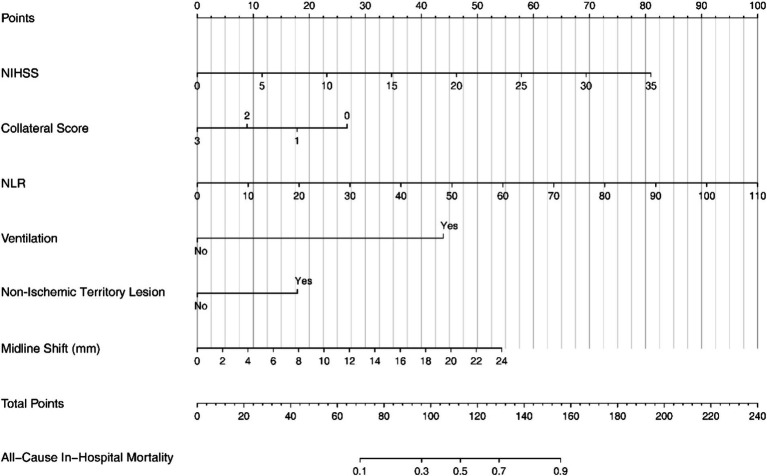
The nomogram for predicting in-hospital mortality in patients with LHI. LHI, large hemisphere infarction.

**Figure 2:**
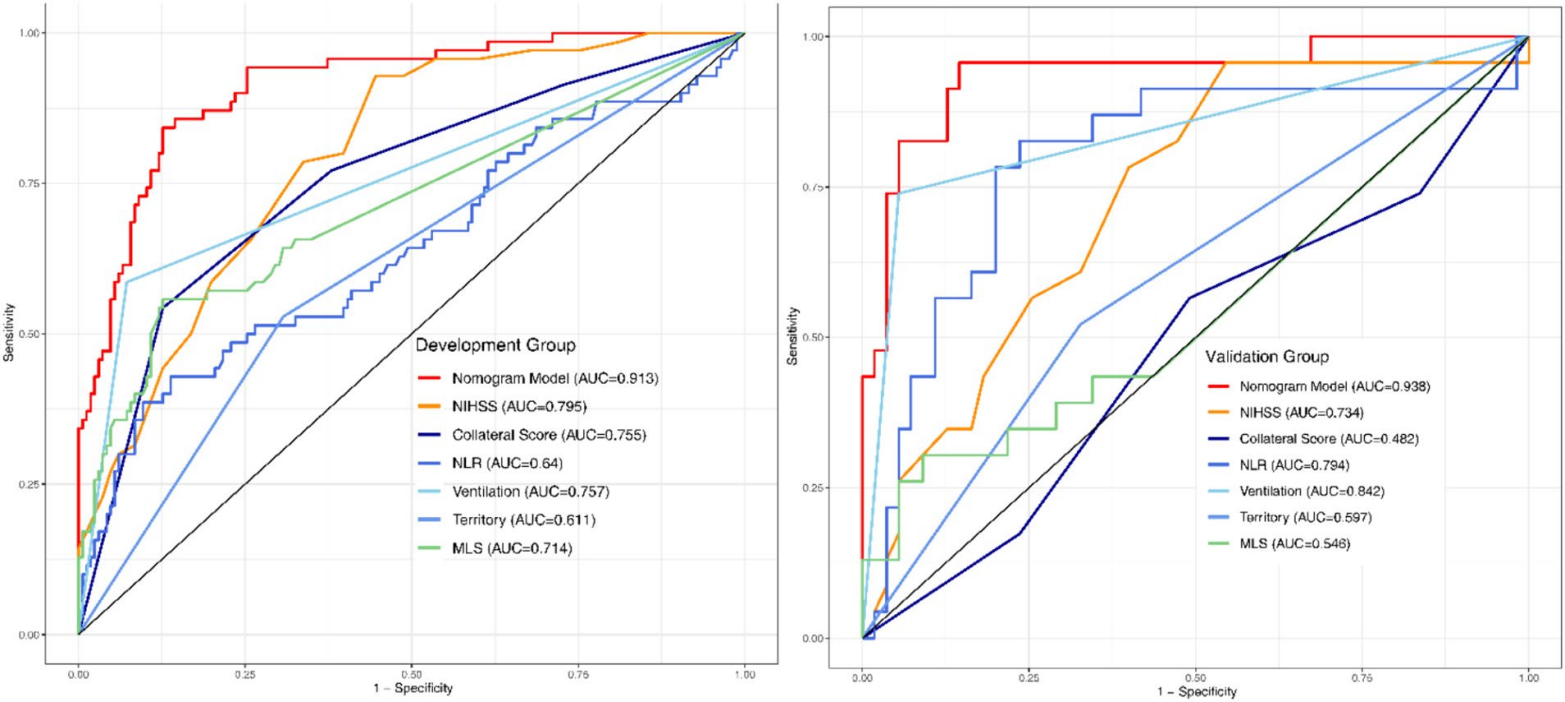
ROC-AUC curves of the development and validation groups. ROC, Receptor operating characteristic curve; AUC, Area under the curve.

**Figure 3:**
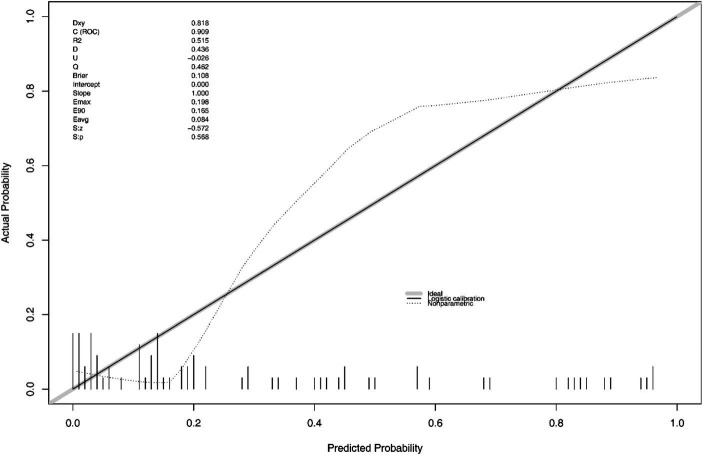
Calibration curve of the nomogram in the validation set.

**Figure 4:**
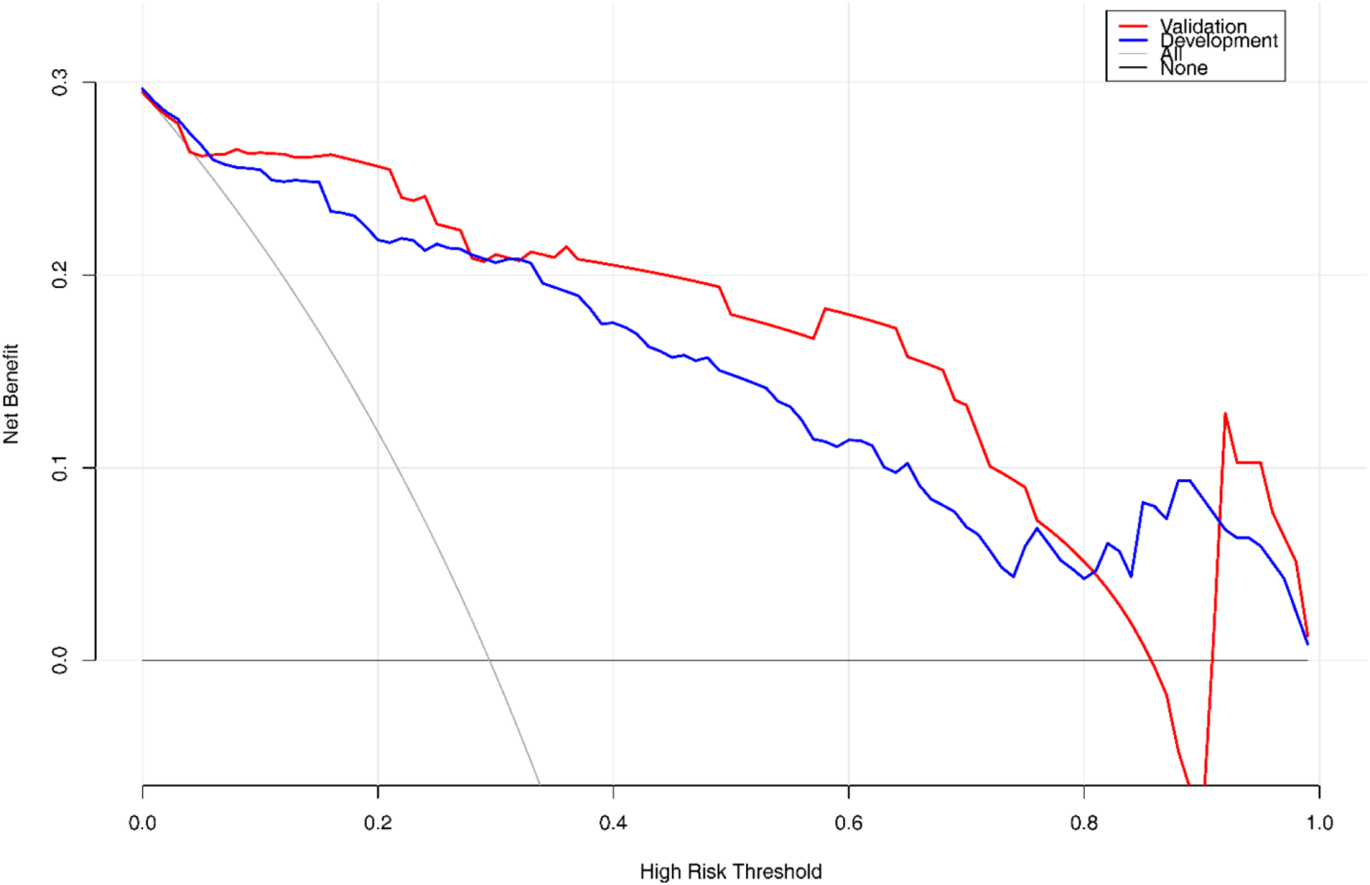
Decision curve analysis of the nomogram model.

The final Logistic Regression model was subjected to Nomogram construction (Figure 1), and ROC curves were generated and AUC values calculated for both the Development Group and the Validation Group (Figure 2). It is evident that the model exhibits excellent discriminative capacity and generalizability. In the Validation Group, calibration curves were generated (Figure 3), demonstrating a high degree of conformity between the calibrated model and actual observations. Lastly, DCA curves were crafted (Figure 4) for both the Development Group and the Validation Group, revealing the model’s remarkable clinical net benefit capability. Within the threshold range of 5 to 85%, the model’s performance surpasses that of clinical net benefit under complete intervention and non-intervention scenarios.

The steps for using the nomogram are to score each sub-item up to the corresponding scale, then add them up, and read the total score on the lower scale bar, which points to the final probability line. The resulting probability is the probability of all-cause mortality during the patient’s hospitalization in this Nomogram.

For example, a 50-year-old male patient diagnosed with large hemispheric stroke was admitted to the Emergency Room with an NIHSS score of 15, a collateral score of 2, an NLR of 20, requiring mechanical ventilation, presenting with a non-ischemic territory lesion, and a midline shift of 6 mm. Using the Nomogram, the points assigned for each parameter are as follows: NIHSS score of 15: 35 points; Collateral score of 2: 10 points; NLR of 20: 18 points; Ventilation: 45 points; Non-Ischemic Territory Lesion: 0 points; Midline Shift of 6 mm: 12 points.

Summing these points, the total is 120. According to the Nomogram, this total corresponds to an all-cause in-hospital mortality probability of approximately 60%.

### 3.4 Models’ comparison

The previous study (5) included solely of MLS, Age, and the logarithmically transformed NLR renders the model insufficiently robust, exhibiting an excessive degree of simplicity. Through the utilization of the NRI and IDI metrics, we have comprehensively surpassed the original model. Comparing to the former one, our model achieved NRI (Continuous) 1.189, 95% CI [0.9588–1.4191]; *p*-value<0.001. IDI 0.2951, 95% CI [0.225–0.3651]; *p*-value<0.001. Which means through the utilization of the NRI and IDI metrics, we have comprehensively surpassed the original model.

## 4 Discussion

LHI imposes a substantial burden on both patients and their family members due to its high mortality. Identifying potential risk factors for in-hospital mortality at an early stage is essential for both patients and clinicians. In our study, the mortality of patients with LHI is 29.6%, which is comparatively low (1). Several factors contribute to this lower mortality rate. First, decompressive hemicraniectomy was performed in some of our patients, leading to a reduction in mortality (8). Second, the participants in our study were elderly, with an average age of 75.67 years. Elderly patients may exhibit brain atrophy, which allows for more space for brain swelling.

Previous study has reported that MBE, pulmonary infection, and hypoalbuminemia are independently associated with a 3-month unfavorable outcome in patients with right-sided large hemisphere infarction (RLHI) (9). Additionally, admission NIHSS>20 and mechanical ventilation within 48 h of admission were independently associated with a poor outcome in very elderly patients with LHI which received medical management only (10). In our study, we found that MLS, ventilation, NLR, NIHSS, collateral score and involve of non-ischemic territory could predict the in-hospital mortality in LHI patients (Figure 1).

We have identified that MLS is a crucial independent factor associated with poor outcome in LHI patients. MLS typically indicates the presence of malignant brain edema (MBE), a significant contributor to early mortality. MBE can lead to irreversible tissue damage, inadequate cerebral blow flow, an impaired blood–brain barrier (BBB), elevated intracranial pressure and brain herniation (11). Li et al. (9) reported that MBE is linked to unfavorable outcome in patients with RLHI independently. Thus, early detection of brain edema is imperative.

In a previous study (6) focused on predicting the risk of MCE after acute LHI involving the anterior circulation, eight independent predictors were identified, including GCS score, NIHSS score, ASPECTS, monocyte count, WBC count, HbA1c level, history of hypertension, as well as a history of hypertension and atrial fibrillation. While this study shed light on the predictive mechanisms of LHI progression to MCE, it did not delve into the exploration of risk prediction mechanisms for in-hospital mortality in patients who did not undergo DHC. To manage elevated intracranial pressure resulting from cerebral edema, hyperosmolar agents such as hypertonic saline and mannitol are commonly employed. However, these therapies require an intact BBB to exert their osmotic effects and may not be effective at the site of edema (12). DHC has been advocated as an effective treatment to reduce MBE-related mortality, but its criteria are stringent. For instance, very elderly patients may not be candidates for this procedure (10). The GAMES-RP trial (13) demonstrated that glibenclamide, when compared to a placebo, significantly reduce mortality at 30 days, although no distinction in mortality rates was observed (13) between days 7 and 90. Therefore, early detection and treatment of brain edema are vital steps to reduce the mortality rates among patients suffering from large-scale cerebral infarctions in the future.

In our study, we also found that mechanical ventilation was another independent predictor for mortality, consistent with previous researches (14, 15) which highlighted that a high mortality rate among patients requiring artificial ventilation. Zhang et al. (16) demonstrated that mechanical ventilation represented an independent risk factor for in-hospital mortality in acute stroke patients (15). On one hand, patients with LHI usually require mechanical ventilation due to severe brain damage, swallowing dysfunction, and impaired consciousness. On the other hand, serious complications such as pneumonia and sepsis may result in respiratory failure, necessitating mechanical ventilations. According to the most recent finding, the outcome is worse for those who need mechanical ventilation on the day of stroke diagnosis compared to those who require it after diagnosis (14). Although the exact mechanism remains unclear, it appears that mechanical ventilation is the underlying factor contributing to this discrepancy. This is attributed to the fact that mechanical ventilation augments the susceptibility to lung infections, ventilator-associated pulmonary injuries, respiratory failure, and additional complications, all of which can substantially heighten the risk of mortality (17).

In the last few years, inflammation has emerged as a pivotal factor in predicting the prognosis of cerebral infarction. In our study, we identified that the NLR was associated with poor outcome of patients with LHI, consistent with previous study that NLR was the highly potential predictor of clinical outcomes (18), additionally, the study also suggested that NLR can be an indicator of brain edema and death in individuals suffering from a large-scale cerebral infarction. Ji et al. (19) also reported that NLR had the exceptional predictive ability for in-hospital mortality following acute myocardial infarction, it appears that inflammatory factors can infiltrate ischemia-damaged tissues, including myocardial and brain tissues, and exert their effects. Prior study (18) postulated that cerebral edema might constitute a crucial mechanism linking systemic inflammation to secondary brain injury and stroke morbidity, a hypothesis with which we concur. Stroke initiates an early disruption of the blood–brain barrier (BBB), permitting the infiltration of peripheral immune cells into injured tissues (20). Inflammations in infarcted regions would rapidly coalesces within a few hours, then occluding the microvascular network, reducing the microvessel blood flow, and exacerbating tissue damage (21). By triggering local inflammation, this process may worsen the existing endothelial damage, thus resulting in further brain injury due to cerebral edema. An experiment revealed that when a vessel is blocked, neutrophils quickly accumulate in the downstream microcirculation veins. This phenomenon, known as downstream microvascular thromboinflammation (DMT), is intensified by neutrophil activation (22). A recent MRI study (23) in mice has lent credibility to the idea that DMT could potentially exacerbate ischemic damage and disrupt the BBB, thereby potentially leading to hemorrhagic transformation. Both studies had suggested that specific substances released by inflammatory cells, such as oxygen species, proteases, cytokines and chemokines could increase the neuronal death. Boisseua et al. (24) found that Neutrophil count predicts poor outcomes after endovascular therapy. Cui et al. (25) also demonstrated that early peripheral neutrophil count after stroke correlates with infarct size and the fatal outcome of LHI patients. Thus, the control of inflammatory cell aggregation and the subsequent inflammatory reactions is imperative in reducing brain edema, preventing bleeding transformation, and decreasing mortality in patients with LHI.

The NIHSS score is a valuable tool for diagnosing and treating clinical cerebral infarction. It assesses the degree of functional impairment in patients with cerebral infarction, including aspects like speech, consciousness, and limb activity. Generally, a higher the score indicates a more severe condition. Previous investigations have shown that NIHSS scores can be utilized to predict the onset of brain edema (26). It has been observed that a high NIHSS score is associated with an unfavorable clinical outcome (9). Consistent with previous studies, our research reveals a correlation between the NIHSS score and mortality. Therefore, healthcare professionals should pay particular attention to patients with higher NIHSS scores.

The Collateral Score (CS) and infarctions involving Non-MCA Perfusion Territories have both demonstrated associations with the mortality of LHI patients. Jo et al. (26) demonstrated that CS was independently associated with malignant brain edema. Other research (27) had similarly found that poor collateral status independently predicts malignant infarction in patients receiving endovascular therapy. In a study conducted by Elijovich et al. (28), a favorable collateral status was associated with smaller infarct volumes and improved clinical outcomes in patients who underwent endovascular recanalization. Collateral circulation represents an existing vascular pathway that can supply blood to target tissue in the event of blockages in the primary vascular channels (29). Consequently, collateral flow is an effective technique for augmenting blood supply to protect the neurons in ischemic areas. When collateral flow is insufficient, irreversible neuronal damage can occur in a matter of minutes (30). The provision of collateral circulation is integral to the development of cerebral ischemia, albeit challenging to measure due to its intricate and narrow pathways (31). The CS serves as a fundamental yet reliable method for evaluating collateral supply and its correlation with smaller infarct volumes. Non-middle cerebral artery infarction usually implies the involvement of other blood vessels, such as the anterior or posterior cerebral artery regions. A previous study (32) had established that anterior cerebral artery involvement is an independent predictor for malignant cerebral edema in LHI patients. Payabvash et al. (33) similarly demonstrated that anterior extension of LHI, which involves the ACA territory and ACA-MCA border zone, independently predicts poor functional outcomes in LHI patients. When the ACA territory is affected in LHI patients, it likely indicates a larger infarction or an occlusion closer to the internal carotid artery, which is often accompanied by reduced hemispheric collateral flow and the presence of edematous brain tissue (34). Nevertheless, alternative perspectives have been presented in some articles. Kürten et al. (35) suggested that patients with infarctions extending beyond the MCA territory have similar likelihoods of positive outcomes as those with solely MCA infarction. Further research is needed on these topics.

Nomogram is a widely popularized visual tool for Logistic regression models in recent years, enabling a clear and concise risk prediction for binary classification tasks. Sun’s article had previously conducted a similar study and discovered that age, NLR, and MLS are the risk factors for mortality, which aligns with our findings (5). However, our study offers three distinct advantages in comparison to the prior research. Firstly, our research has a larger sample size and a more comprehensive population representation than the previous investigation. The earlier study had a sample size of 158 participants, ranging in age from 53 to 71 years. In contrast, our study encompassed 314 individuals, aged between 32 and 104 years. Secondly, our research has identified a broader range of predictive factors that can be easily accessed from routine clinical practice, thus enhancing their practical utility. Previous research has highlighted the challenges in obtaining predictive factors, such as the requirement for logarithmic values in the case of NLR. Moreover, in contrast to prior research, this study utilized a variant of the random forest-based artificial intelligence method known as Boruta for variable selection. Unlike the conventional approach employed in the study mentioned above, which involved a sequential process of univariate regression followed by multivariate regression, our method accounts for interactions between variables, resulting in more robust outcomes.

Despite our best efforts to mitigate potential constraints, like applying AI technique Boruta algorithm (7). Despite which made a key role in variable selection, our study still exhibits certain limitations. Firstly, it is important to acknowledge that the study is based on a single-center sample. Secondly, while our prediction tool demonstrated high performance, it has only been internally validated. Future research should prioritize the investigation of subgroups and their clinical implications to enhance the generalizability of our findings.

In conclusion, our research demonstrated that MLS, ventilation, NLR, NIHSS, collateral score and non-ischemic territory are reliable indicators for predicting in-hospital mortality in LHI patients. Our prediction model can serve as a useful tool for clinicians in guiding appropriate treatment strategies for patients with ischemic stroke.

The limitations of our Nomogram and future developments include:

1. Need for external validation: our model requires validation with larger and more diverse external datasets to assess its generalizability. This includes data from populations undergoing vascular recanalization treatments.
2. Existing scores: there are existing scores for EDEMA developed in South Korea, the USA, and China (36). Although these scores need improvement in terms of generalizability, especially in populations without access to endovascular treatment, they provide a foundation for further research.
3. Development of a standardized score: current models, such as the MBE, EDEMA and modified versions of EDEMA in China, focus on different aspects, like the weight of imaging indicators. There is a need to develop a standardized, globally applicable scoring system for EDMA and its complications, suitable for acute stroke patients of all ethnicities.

## 5 Conclusion

Our study has successfully employed NIHSS score, Collateral Score, NLR, mechanical ventilation, non-MCA territorial involvement in patients, and midline shift to construct a highly accurate, user-friendly nomogram for in-hospital mortality prediction in LHI patients. This nomogram provides valuable reference information for future investigations into the prognosis of LHI patients and death prevention. Furthermore, it addresses the shortcomings of previous research.

## Data availability statement

The raw data supporting the conclusions of this article will be made available by the authors, without undue reservation.

## Ethics statement

The studies involving humans were approved by the Ethics Review Board of the Third Affiliated Hospital of Soochow University (Approval Number: 2023-S-080). The studies were conducted in accordance with the local legislation and institutional requirements. The ethics committee/institutional review board waived the requirement of written informed consent for participation from the participants or the participants’ legal guardians/next of kin because it’s a retrospective cohort, so there’s no need to sign the informed consents.

## Author contributions

JD: Supervision, Validation, Writing – original draft, Writing – review & editing. XM: Writing – original draft, Writing – review & editing, Project administration, Conceptualization, Data curation, Formal analysis, Investigation, Software, Validation. WH: Data curation, Formal analysis, Software, Supervision, Validation, Writing – original draft, Writing – review & editing. CY: Data curation, Formal analysis, Investigation, Writing – original draft, Writing – review & editing, Methodology. GX: Conceptualization, Data curation, Resources, Writing – original draft, Writing – review & editing. YW: Data curation, Formal analysis, Investigation, Writing – original draft, Writing – review & editing. SS: Conceptualization, Data curation, Formal analysis, Funding acquisition, Investigation, Methodology, Project administration, Resources, Software, Supervision, Validation, Visualization, Writing – original draft, Writing – review & editing. ML: Writing – original draft, Writing – review & editing, Data curation, Methodology, Conceptualization, Formal analysis, Funding acquisition, Investigation, Project administration, Resources, Software, Supervision, Validation, Visualization. YR: Conceptualization, Data curation, Formal analysis, Funding acquisition, Investigation, Methodology, Project administration, Resources, Software, Supervision, Validation, Visualization, Writing – original draft, Writing – review & editing.
