# Supplementary material for: Validation and refinement of a predictive nomogram using artificial intelligence: assessing in-hospital mortality in patients with large hemispheric cerebral infarction
Source: Front Neurol. 2024 Jun 25;15:1398142. doi: 10.3389/fneur.2024.1398142 (PMC11231922; doi:10.3389/fneur.2024.1398142)
Supplement: Supplementary file 1 [file Table_1.DOCX]

Supplementary Material

Validation and Refinement of a Predictive Nomogram Using Artificial Intelligence: Assessing In-Hospital Mortality in Patients with Large Hemispheric Cerebral Infarction

**Jian Ding^1†^, Xiaoming Ma^2†^, Wendie Huang^1†^, Chunxian Yue^1†^, Geman Xu^3^, Yumei Wang^1^, Shiying Sheng^1^, Meng Liu^1*^, Yi Ren^1*^**

^1^Department of Neurology, The Third Affiliated Hospital of Soochow University, Changzhou, 213000, PR China

^2^ Suzhou Hospital, Affiliated Hospital of Medical School, Nanjing University, Suzhou, 215000, PR China

^3^Department of Neurology, Affiliated Fuyang People’s Hospital of Anhui Medical University, Fuyang, 236000, PR China

*** Correspondence:
Yi Ren**

**rylemon18000@163.com**

Meng Liu

lm145@163.com

**It should be note that Yi Ren is the first Corresponding Author.**

# Supplementary Table 1

**Supplementary Table 1. Demographic characteristics & clinical information of Development & Validation Group.**

| Characteristics | All (n=314) | Development Group （n = 236) | Validation Group (n = 78) | *p*-value |
| --- | --- | --- | --- | --- |
| Age | 75.67 ± 12.28 | 75.59 ± 12.16 | 75.91 ± 12.71 | 0.844 |
| Glascow Coma Score | 9.90 ± 3.56 | 9.88 ± 3.62 | 9.96 ± 3.40 | 0.856 |
| ASPECTS | 5.32 ± 3.08 | 5.22 ± 3.08 | 5.59 ± 3.08 | 0.365 |
| NIHSS score | 16.81 ± 6.56 | 16.65 ± 6.58 | 17.31 ± 6.49 | 0.442 |
| Collateral Score | 1.49 ± 1.08 | 1.47 ± 1.09 | 1.54 ± 1.04 | 0.629 |
| WBC | 28.93 ± 74.04 | 31.23 ± 78.69 | 21.97 ± 57.60 | 0.339 |
| Neutrophil count | 8.91 ± 3.67 | 8.95 ± 3.61 | 8.79 ± 3.88 | 0.741 |
| Lymphocyte count | 1.77 ± 2.57 | 1.88 ± 2.76 | 1.44 ± 1.87 | 0.188 |
| NLR | 9.20 ± 9.00 | 9.21 ± 9.67 | 9.19 ± 6.62 | 0.991 |
| Fibrinogen level | 3.94 ± 2.33 | 3.88 ± 1.95 | 4.14 ± 3.21 | 0.397 |
| SBP | 149.77 ± 23.30 | 149.83 ± 23.97 | 149.58 ± 21.28 | 0.933 |
| DBP | 83.03 ± 14.93 | 83.05 ± 14.94 | 82.97 ± 15.00 | 0.969 |
| APACHE II | 13.20 ± 5.50 | 13.03 ± 5.65 | 13.72 ± 5.01 | 0.342 |
| Midline Shift (mm) | 3.41 ± 5.28 | 3.41 ± 5.37 | 3.41 ± 5.06 | 0.328 |
| Sex |  |  |  |  |
| Female | 156 (49.68) | 113 (47.88) | 43 (55.13) | 0.328 |
| Male | 158 (50.32) | 123 (52.12) | 35 (44.87) |  |
| TOAST Classification |  |  |  |  |
| Large-artery atherosclerosis | 171 (54.46) | 129 (54.66) | 42 (53.85) | 1.000 |
| Cardioembolism | 143 (45.54) | 107 (45.34) | 36 (46.15) |  |
| Consciousness disorders |  |  |  |  |
| No | 110 (35.03) | 86 (36.44) | 24 (69.23) | 0.439 |
| Yes | 204 (64.97) | 150 (63.56) | 54 (25.64) |  |
| History of hypertension |  |  |  |  |
| No | 72 (22.93) | 52 (22.03) | 20 (25.64) | 0.616 |
| Yes | 242 (77.07) | 184 (77.97) | 58 (74.36) |  |
| History of diabetes mellitus |  |  |  |  |
| No | 200 (63.69) | 150 (63.56) | 50 (64.10) | 1.000 |
| Yes | 114 (36.31) | 86 (36.44) | 28 (35.90) |  |
| Atrial fibrillation |  |  |  |  |
| No | 177 (56.37) | 135 (57.20) | 42 (53.85) | 0.699 |
| Yes | 137 (43.63) | 101 (42.80) | 36 (46.15) |  |
| Ventilation |  |  |  |  |
| No | 241 (76.75) | 183 (77.54) | 58 (74.36) | 0.673 |
| Yes | 73 (23.25) | 53 (22.46) | 20 (25.64) |  |
| History of stroke |  |  |  |  |
| No | 226 (71.97) | 164 (49.49) | 62 (79.49) | 0.119 |
| Yes | 88 (28.03) | 72 (30.51) | 16 (20.51) |  |
| Smoking history |  |  |  |  |
| No | 256 (81.53) | 195 (82.63) | 61 (78.21) | 0.481 |
| Yes | 58 (18.47) | 41 (17.37) | 17 (21.79) |  |
| Drink history |  |  |  |  |
| No | 271 (86.31) | 207 (87.71) | 64 (82.05) | 0.284 |
| Yes | 43 (13.69) | 29 (12.29) | 14 (17.95) |  |
| Pneumonia |  |  |  |  |
| No | 97 (30.89) | 72 (30.51) | 25 (32.05) | 0.909 |
| Yes | 217 (69.11) | 164 (69.49) | 53 (67.95) |  |
| UTI |  |  |  |  |
| No | 283 (90.13) | 214 (90.68) | 69 (88.46) | 0.726 |
| Yes | 31 (9.87) | 22 (9.32) | 9 (11.54) |  |
| Gastrointestinal bleeding |  |  |  |  |
| No | 296 (94.27) | 220 (93.22) | 76 (97.44) | 0.268 |
| Yes | 18 (5.73) | 16 (6.78) | 2 (2.56) |  |
| Hemorrhagic transformation |  |  |  |  |
| No | 236 (75.16) | 178 (75.42) | 74 (79.57) | 0.303 |
| Yes | 78 (24.84) | 59 (26.70) | 19 (20.43) |  |
| Seizure |  |  |  |  |
| No | 303 (96.50) | 226 (95.76) | 77 (98.72) | 0.381 |
| Yes | 11 (3.50) | 10 (4.24) | 1 (1.28) |  |
| Admission anisocoria |  |  |  |  |
| No | 263 (83.76) | 196 (83.05) | 67 (85.90) | 0.679 |
| Yes | 51 (16.24) | 40 (16.95) | 11 (14.10) |  |
| Admission Gaze deviation |  |  |  |  |
| No | 160 (50.96) | 121 (51.27) | 39 (50.00) | 0.949 |
| Yes | 154 (49.04) | 115 (48.73) | 39 (50.00) |  |
| Lesion side |  |  |  |  |
| Left | 149 (47.45) | 110 (46.61) | 39 (50.00) | 0.697 |
| Right | 165 (52.55) | 126 (53.29) | 39 (50.00) |  |
| Infarction Involving Non-MCA Perfusion Territories |  |  |  |  |
| No | 196 (62.42) | 148 (62.71) | 48 (61.54) | 0.960 |
| Yes | 118 (37.58) | 88 (37.29) | 30 (38.46) |  |
| Lateral Ventricular Compression |  |  |  |  |
| No | 136 (43.31) | 102 (43.22) | 34 (43.59) | 1.000 |
| Yes | 178 (56.69) | 134 (56.78) | 44 (56.41) |  |
| Basalcistern effacement |  |  |  |  |
| No | 231 (73.57) | 173 (73.31) | 58 (74.36) | 0.972 |
| Yes | 83 (26.43) | 63 (26.69) | 20 (25.64) |  |
| Death |  |  |  |  |
| No | 221 (70.38) | 166 (70.34) | 55 (70.51) | 1 |
| Yes | 93 (29.62) | 70 (29.66) | 23 (29.49) |  |

Note: No statistically significant differences were existed in all the variables and outcome between the Development Group and the Validation Group.
